# On efficient modelling of radical production in cavitation assisted reactors

**DOI:** 10.1016/j.ultsonch.2024.106833

**Published:** 2024-03-02

**Authors:** Suat Canberk Ozan, Pascal Jan Muller, Jan Hendrik Cloete

**Affiliations:** a Process Technology Department, SINTEF Industry, S.P. Andersens veg 15B, NO-7031 Trondheim, Norway; b Department of Chemical Engineering, Norwegian University of Science and Technology (NTNU), N-7491 Trondheim, Norway; c Department of Mechanical Engineering, ETH Zurich, D-MAVT Leonhardstrasse 21, 8092 Zurich, Switzerland

**Keywords:** Cavitation, Radical production, Gibbs minimization, Single bubble dynamics, Numerical simulations, Reaction modelling

## Abstract

Process intensification by cavitation is gaining widespread attention due to the benefits that the intense bubble collapse conditions can provide, yet, several knowledge gaps exist in the modelling of such systems. This work studies the numerical prediction of single bubble dynamics and the various approaches that can be employed to estimate the changes in the chemical composition of cavitating bubbles. Specific emphasis is placed on the prediction of the radical production rates during bubble collapse and the computational performance, with the aim of coupling the single bubble dynamics to flow models for reactor hydrodynamics. The results reveal that the choice of chemical reaction approach has virtually no effect on the bubble dynamics, whereas the predicted radical production rates can differ substantially. It is found that evaluating the radical production only on temperature peaks, an approach commonly followed in literature, may result in the most erroneous estimations (on average 12.8 times larger than those of the full kinetic model), while a simplified kinetic model yields more accurate predictions (2.3 times larger) at the expense of increased computational times. Continuous evaluation of the bubble content by assuming equilibrium when the bubble temperature is above a certain threshold (≈1500K) is shown to be capable of predicting total radical production values close to those estimated by solving the kinetics of a detailed reaction model (19.8% difference), as well as requiring only 22.2% more computational costs compared to simulations without chemical reaction modelling. Such an equilibrium approach is therefore recommended for future studies aiming to couple flow simulations with single bubble dynamics to accurately predict radical production rates in cavitation devices, involving numerous bubbles following different flow trajectories. Furthermore, an algebraic expression that successfully approximates the full kinetic simulation results is proposed as a function of the initial nucleus size and the time integral of the liquid pressure when it is under vapor pressure. Such a model can be applied in modelling efforts that do not require local instantaneous radical concentrations, and paves the way for efficient closure modelling of radical production in CFD simulations of hydrodynamic reactors.

## Highlights

• Dynamics and chemistry of a cavitating bubble are studied.
• Radical OH production is found to correlate strongly with maximum bubble size.
• Kinetic and equilibrium-based reaction models are compared.
• Assuming equilibrium above 1500 K is shown to be optimal for flow simulations.
• An algebraic expression that accurately estimates radical production is proposed.

## 1 Introduction

Historically, cavitation has been seen as a phenomenon that must be avoided especially due to the harm it may inflict on hydraulic machinery [1] and erosion concerns [2]. Although this point of view is still valid for many applications, it has also been shown that both acoustically and hydrodynamically induced cavitation can be exploited for process intensification, e.g., in wastewater treatment [3], hydrogen production [4] and biogas production [5], or for improved technical procedures, e.g., in medicine [6] and nanoparticle synthesis [7].

Under acoustic cavitation conditions, the dynamics of a single cavitating bubble and the changes in its chemical composition have been extensively investigated in the literature [8], [9], [10], [11], [12], [13], [14]. Although these investigations shed light on the nature of cavitation bubbles and improved the understanding of the phenomenon considerably, process intensification efforts require the consideration of the cavitation phenomenon in large scale cavitation devices. For the theoretical analysis of such systems, physics from various length and time scales, ranging from the nucleation and dynamics of individual cavitating bubbles to overall flow behavior (e.g., turbulence and pressure drop), must be simultaneously taken into account. Together with the complex geometries of the orifices, venturis, or vortex devices employed as cavitation devices, these considerations render computational fluid dynamics (CFD) tools a viable option for the simulation of hydrodynamic cavitation configurations. However, these extensive requirements also bring a trade-off between the overall complexity (and consequent accuracy) of the model and the computational costs. Studies investigating cavitation in flow devices [15], [16], [17] have typically incorporated Eulerian cavitation closure models [18], [19], [20] to reduce the computation times. Although these closures are computationally inexpensive and decent at estimating when and where cavitation occurs in the domain, they have several drawbacks: They stem from a significantly simplified version of the Rayleigh-Plesset equation [21] where surface tension, viscous and some inertial effects are neglected, and indirectly assume that the changes in the water content within the bubble is given through the momentum balance without describing the phenomenon through a mass transfer equation across the bubble interface. Furthermore, the Rayleigh-Plesset equation itself neglects the impact of the shockwaves emerging during cavitation, which can alter the cavitation behavior considerably. For applications, like wastewater treatment, where the radical generation during the collapse of the cavitation bubble is the critical phenomena driving the process, the accurate representation of bubble dynamics plays a key role. Existing Eulerian cavitation closure models are insufficient for investigating such problems, since they provide information only about how much water vapor is generated and where, and no information about the bubble conditions during collapse, which drives the production of radical species.

On the other end of the spectrum, to avoid the discrepancies inherent to the Eulerian cavitation closures currently available in the CFD framework, several works attempted to simplify the flow modelling (and the flow geometry) and placed the emphasis on better representation of the bubble dynamics and changes in the bubble chemical composition. Wang and Brennen [22] studied cavitating flows through a converging–diverging nozzle by combining the continuity and the momentum equations for a bubbly flow with a formulation of the Rayleigh-Plesset equation without considering any reaction modelling. Krishnan et al. [23] employed a detailed model considering momentum, mass, and energy balances for the bubble dynamics, originated from Storey and Szeri [11]’s work, in combination with scaling arguments and Bernoulli equation for the estimation of the bulk pressure field to evaluate the operating conditions’ effect on their orifice plate configuration. They employed an 8 reaction kinetic scheme to investigate the radical production. Sharma et al. [24] studied a similar orifice plate-based hydrodynamic cavitation configuration to reveal the effects of orifice diameter to plate diameter ratio, initial nucleus size, and inlet pressure on the radical production rate and collapse conditions using an extensive 45 reaction kinetic scheme originally proposed by Toegel et al. [13]. Kumar et al. [25] assumed that the mean flow expands linearly after the orifice to analyze the effect of operating conditions on the cavitating flow regimes. Extending the work of Kumar et al. [25], Capocelli et al. [26] included changes in chemical composition of the cavitation bubbles in their model and presented radical production estimates in a venturi channel by evaluating the equilibrium composition of the bubble at its peak temperature through minimization of Gibbs energy instead of incorporating a time dependent kinetic model.

The trade-off between the computational cost and the predictive power of the hydrodynamic cavitation models limits the modelling efforts to either emphasize the resolution of the flow field and complex geometries involved or the resolution of the single bubble level physics and chemistry. To be able to fully incorporate phenomena from all scales involved, an approach that can be coupled to CFD simulations while accurately representing the cavitating bubble dynamics and chemical reactions at low computational costs is required. When a single bubble is considered under acoustic cavitation conditions these costs are not of significant concern. However, this is not the case for the hydrodynamic cavitation configurations where a significant amount of bubbles cavitate simultaneously, but under different conditions due to their various flow trajectories. Thus, this work investigates the reactive single bubble dynamics and the impact of different reaction modelling approaches that could be employed to represent the chemical changes in the bubble, with the goal of identifying the optimal approach that will yield accurate estimates of the bubble content with the highest computational efficiency. In addition, the potential for modelling the amount of radical production during the collapse of a cavitation bubble using an algebraic expression based on operational inputs, and without the need for detailed modelling of the bubble dynamics, is evaluated.

Section 2 presents the mathematical models employed in the analysis for the bubble dynamics (Section 2.1) and the different kinetic and equilibrium methods used for modelling reactions (Section 2.2). The numerical procedure for the two-step coupled dynamics-reaction solver is described in Section 3. The results and discussion are grouped under three subsections: Section 4.1 analyses the dynamics and chemistry of a reactive single bubble subject to a pressure variation cycle. Section 4.2 focuses on the predictive power and computational expense of the different reaction modelling approaches investigated and recommends an optimal approach for use in detailed cavitation assisted reactor modelling. Section 4.3 proposes an algebraic model that predicts the amount of radical produced during cavitation without requiring the resolution of the bubble dynamics. Finally, the conclusions drawn throughout the study are summarized in Section 5.

## 2 Mathematical model

The modelling efforts are caterogized under two titles. Section 2.1 presents the single bubble dynamics equations governing the bubble radius, temperature, and pressure under cavitation conditions, as well as the phase change of water molecules due to cavitation. Section 2.2 focuses on the modelling of changes in the chemical composition of the cavitating bubble due to the reactions taking place under extreme temperatures and pressures achieved during the bubble collapse. The resulting sets of equations are then coupled to form the reactive single bubble model.

### 2.1 Single bubble dynamics (SBD)

The single bubble model covers the dynamics of a spherical bubble with a non-deformable spherical interface subject to an applied pressure field. Changes in the applied pressure field results in its growth or shrinkage of the bubble, either due to the force balance on the bubble or the transfer of $H_{2}O$ molecules between the bubble and the surrounding liquid. During the collapse of the cavitation bubble, rapid dynamics can lead to extremely high bubble temperatures and pressures, as well as shockwaves. Under such conditions, the chemical composition of the bubble may change significantly, resulting in formation of radical molecules that eventually leave the bubble and react with the other molecules present in the liquid phase, for example, incalcitrant micropollutants when applied to wastewater treatment.

By assuming the cavitation bubble always remains spherical and has uniform properties, changes in its size can be estimated through the Rayleigh-Plesset equation [21]:

(1) $$\overset{¨}{R}R+\frac{3}{2}{\overset{˙}{R}}^{2}=\frac{p_{B}-p_{L}}{\rho_{L}}-4\nu_{L}\frac{\overset{˙}{R}}{R}-\frac{2\gamma }{\rho_{L}R}$$

where *R* is the bubble radius, *p* is pressure, and ρ,ν and γ are the density, the kinematic viscosity and the surface tension, respectively. The subscripts *B* and *L* denote the bubble and the surrounding liquid properties and the dot operator stands for the total time derivative. Although Eq. (1) successfully represents the growth/shrinkage of a bubble under milder conditions, it does not consider the shockwaves generated during cavitation, which may have an immense effect on the resulting bubble size [27]. Thus, to account for the shockwaves, the Keller-Miksis equation [28] is employed instead:

(2) $$\overset{¨}{R}R\left(1-\frac{\overset{˙}{R}}{a_{L}}\right)+\frac{3}{2}{\overset{˙}{R}}^{2}\left(1-\frac{\overset{˙}{R}}{3a_{L}}\right)=\frac{p_{B}-p_{L}}{\rho_{L}}\left(1+\frac{\overset{˙}{R}}{a_{L}}\right)+\frac{R}{\rho_{L}a_{L}}\overset{˙}{p_{L}}-4\nu_{L}\frac{\overset{˙}{R}}{R}-\frac{2\gamma }{\rho_{L}R}$$

where $a_{L}$ is the speed of sound within the liquid phase. The bubble dynamics were found to be qualitatively independent of the selection of the gas phase equation of state in the literature [10], [29]. Consequently, in the current work, the bubble pressure is determined through the ideal gas law. The bubble nuclei are assumed to consist of argon at initiation. Mass transfer of non-condensable species, such as argon, to and from the bubble is expected to have a limited effect and is therefore neglected in the model. On the other hand, mass transfer of $H_{2}O$ is important to model. For the mass transfer across the interface and the bubble energy balance, the formulations proposed by Toegel et al. [30] are adopted. The rate of transfer across the interface is given by

(3) $${\overset{˙}{N}}_{w}=4\pi R^{2}D_{w}\frac{c_{\mathrm{wR}}-\frac{N_{w}}{V_{B}}}{l_{\mathrm{diff},w}}$$

where *N* is the amount of molecules inside the bubble in $\mathrm{mol},c_{R}$ is the molar concentration at the liquid side of the interface, *D* is the molecular diffusivity, $V_{B}$ is the bubble volume, and $l_{\mathrm{diff}}$ is the diffusive length. The subscript *w* stands for the water vapor properties. The vapor concentration at the liquid side is assumed to be at equilibrium, i.e., $c_{\mathrm{wR}}=p_{\mathrm{vap}}/R_{g}/T_{L}$, where $p_{\mathrm{vap}}$ is the vapor pressure, $T_{L}$ is the liquid temperature, and $R_{g}$ is the universal gas constant. In the estimation of the diffusive length, it is assumed that the bubble can be treated as a hot homogeneous core surrounded by a cold boundary layer that is in thermal equilibrium with the liquid, yielding $l_{\mathrm{diff}}=\min\left(\sqrt{\mathrm{RD}/\overset{˙}{R}},R/\pi \right)$. The detailed derivation and discussion of the approach can be found in Toegel et al. [30]. An energy balance over the bubble yields the governing equation for the bubble temperature as:

(4) $${\overset{˙}{T}}_{B}=\frac{1}{c_{v,B}}\left(4\pi R^{2}\lambda_{B}\frac{T_{L}-T_{B}}{l_{\mathrm{th}}}-4\pi R^{2}\overset{˙}{R}p_{B}+(h_{w}-e_{w}){\overset{˙}{N}}_{w}\right)$$

where $c_{v}$ is the heat capacity at constant volume, λ is the thermal conductivity, *h* is the enthalpy, and *e* and $l_{\mathrm{th}}$ are the internal energy and thermal diffusive length, respectively. In analogy with $l_{\mathrm{diff}},l_{\mathrm{th}}=\min\left(\sqrt{R\kappa /\overset{˙}{R}},R/\pi \right)$ according to the cold-boundary layer assumption [30], where κ is thermal diffusivity of the gas mixture.

The required closures for Eqs. (2), (3), (4) and the temperature dependencies of the physical properties are presented in the Appendix.

### 2.2 Chemical reactions

At the extreme conditions achieved during cavitation, the vapor molecules inside the bubble are no longer stable and radicals are generated. The chemical composition of the bubble can then be estimated either by directly including the reaction kinetics [8], [13], [23], [31] in the single bubble model or by evaluating the equilibrium compositions under these extreme conditions [26], [32], [33], [34]. The ’kinetic’ approach is the more computationally-expensive one as the reaction kinetics typically require orders of magnitude smaller time scales to be resolved in comparison to what is needed to solve for the bubble dynamics in Eqs. (2), (3), (4). Therefore, the equilibrium based approaches has been an attractive alternative in the literature, especially in the analysis of systems involving several cavitating bubbles such as hydrodynamic cavitation devices. In this approach, the equilibrium composition of the bubble is determined through minimization of the Gibbs free energy once at the peak bubble temperature [26], [32], [33], [34]. Alternatively, the equilibrium compositions can be continuously evaluated multiple times when the bubble temperature is above a certain threshold. Here, the former method will be referred to as the peak-evaluation approach, and the latter as the equilibrium approach. The peak-evaluation approach assumes that the radicals produced under peak conditions leave the bubble instantaneously without any further kinetics or equilibrium evaluation. Although this approach is computationally more advantageous, it is not capable of rendering the further chemical reactions occurring during the collapse as it depends on a single instantaneous equilibrium evaluation. In reality, the radicals would be produced and transferred to the liquid phase continuously at sufficiently high temperatures for longer time-spans, and in the meanwhile, the reverse reactions start to consume some of the produced radicals, as the bubble cools. In this work both kinetics and equilibrium based approaches are tested and the resulting radical generation rates are compared.

#### 2.2.1 Equilibrium based approaches

The equilibrium and the peak-evaluation approaches rely on the minimization of the Gibbs free energy inside the bubble. During the minimization, the bubble temperature and pressure is assumed to be constant, either when temperature peaks or when it is above a certain threshold depending on the approach employed, and an optimization problem with the species concentrations as the free parameters is solved [35]. The problem is subject to constraints stemming from conservation of elements present in the system. For the evaluation of the equilibrium compositions, the ’equilibrate’ function of the open source toolbox Cantera [36] is employed.

#### 2.2.2 Kinetics based approaches

Kamath et al. [8] theoretically investigated the chemical changes in a cavitating bubble (initially filled with argon) by considering a 19 reaction kinetic system (Table 1). They concluded that the reactions 3,4,7 and 8 are the most influential ones in the system. In this work both the full kinetic system, i.e., the 19 reactions presented in Table 1, and the simplified one consisting of these 4 reactions are considered.

**Table 1:** Reaction mechanism for a cavitating bubble initially filled with argon. Subscripts *f* and *b* stand for the forward and the backward reactions, and the dots specify the radical species. The units for the kinetic parameters are given in accordance with concentration unit of $\mathrm{mol}/{\mathrm{cm}}^{3}$, and activation energies and heat of reaction in cal/mol. Adapted from Kamath et al. [8] and Dehane et al. [37].

No.	Reaction	$A_{f}^{*}$	$\beta_{f}$	$E_{a,f}$	$A_{b}^{*}$	$\beta_{b}$	$E_{a,b}$	$\Delta H_{\mathrm{rxn}}$
1	$H^{•}+O_{2}⇌O+{}^{•}\mathrm{OH}$	1.92E14	0	16440	7.18E11	0.36	−679	16540
2	$O+H_{2}⇌H^{•}+{}^{•}\mathrm{OH}$	5.08E4	2.67	6292	2.64E4	2.65	4462	1970
3	${}^{•}\mathrm{OH}+H_{2}⇌H^{•}+H_{2}O$	2.18E8	1.51	3430	1.02E9	1.51	18620	−15400
4	${}^{•}\mathrm{OH}+{}^{•}\mathrm{OH}⇌O+H_{2}O$	2.1E8	1.4	397	2.21E9	1.4	16628	17370
5	$H_{2}+M⇌H^{•}+H^{•}+M$	4.58E19	−1.4	104400	2.45E20	−1.78	960	106330
	$\eta =H_{2}/2.5/H_{2}O/16$							
6	$O+O+M⇌O_{2}+M$	6.17E15	−0.5	0	1.58E17	−0.5	118182	−120910
	$\eta =H_{2}/2.5/H_{2}O/16$							
7	$O+H^{•}+M⇌{}^{•}\mathrm{OH}+M$	4.72E18	−1	0	4.66E17	−0.65	101660	−104360
	$\eta =H_{2}O/5$							
8	$H^{•}+{}^{•}\mathrm{OH}+M⇌H_{2}O+M$	2.25E22	−2	0	1.96E22	−1.62	118600	−121720
	$\eta =H_{2}/2.5/H_{2}O/16$							
9	$H^{•}+O_{2}+M⇌{\mathrm{HO}}_{2}^{•}+M$	2.00E15	0	−1000	2.46E15	0	48290	−49000
	$\eta =H_{2}/2.5/H_{2}O/16$							
10	${\mathrm{HO}}_{2}^{•}+H^{•}⇌H_{2}+O_{2}$	6.63E13	0	2126	2.19E13	0.28	56420	−57340
11	${\mathrm{HO}}_{2}^{•}+H^{•}⇌{}^{•}\mathrm{OH}+{}^{•}\mathrm{OH}$	1.69E14	0	874	1.08E11	0.61	36220	−38820
12	${\mathrm{HO}}_{2}^{•}+O⇌{}^{•}\mathrm{OH}+O_{2}$	1.81E13	0	−400	3.1E12	0.26	51832	−55470
13	${\mathrm{HO}}_{2}^{•}+{}^{•}\mathrm{OH}⇌H_{2}O+O_{2}$	1.45E16	−1	0	2.18E16	−0.72	69181	−72830
14	${\mathrm{HO}}_{2}^{•}+{\mathrm{HO}}_{2}^{•}⇌H_{2}O_{2}+O_{2}$	3E12	0	1387	4.53E14	−0.39	39140	−41950
15	$H_{2}O_{2}+M⇌{}^{•}\mathrm{OH}+{}^{•}\mathrm{OH}+M$	1.2E17	0	45500	9E11	0.90	−6062	52130
	$\eta =H_{2}/2.5/H_{2}O/16$							
16	$H_{2}O_{2}+H^{•}⇌H_{2}O+{}^{•}\mathrm{OH}$	3.2E14	0	8960	1.14E9	1.36	75870	−69600
17	$H_{2}O_{2}+H^{•}⇌H_{2}+{\mathrm{HO}}_{2}^{•}$	4.82E13	0	7948	1.41E11	0.66	24480	−15380
18	$H_{2}O_{2}+O⇌{}^{•}\mathrm{OH}+{\mathrm{HO}}_{2}^{•}$	9.55E6	2	3970	4.62E3	2.75	18435	−13420
19	$H_{2}O_{2}+{}^{•}\mathrm{OH}⇌H_{2}O+{\mathrm{HO}}_{2}^{•}$	1E13	0	1800	2.8E13	0	32790	−30780

In either case, the rate of each reaction can be expressed as

(5) $$r=A_{f}^{*}T_{B}^{\beta_{f}}\exp\left(\frac{-E_{a,f}}{R_{g}T_{B}}\right)\overset{N_{R}}{\underset{i=1}{\prod }}c_{i}^{\mu_{i}}-A_{b}^{*}T_{B}^{\beta_{b}}\exp\left(\frac{-E_{a,b}}{R_{g}T_{B}}\right)\overset{N_{P}}{\underset{i=1}{\prod }}c_{i}^{\mu_{i}}$$

where $A^{*}$ is the collision frequency, $E_{a}$ is the activation energy, β shows the explicit temperature dependence of $A^{*}$, and μ is the stoichiometric coefficient of a species in the reaction. Subscripts *f* and *b* denote the forward and the backward reaction, respectively. $N_{R}$ and $N_{P}$ are the number of reactants and products in the reaction, respectively. The rate of generation/consumption of species *i* through a single reaction is then given as

(6) $$\frac{{\mathrm{dc}}_{i}}{\mathrm{dt}}=\left\{\begin{array}{cc} -r\mu_{i}, & \mathrm{for}\;\mathrm{reactants}\\ r\mu_{i}, & \mathrm{for}\;\mathrm{products} \end{array}\right)$$

and the total rate of generation/consumption of species *i* is determined through summation of Eq. (6) over all the reactions considered in the model.

Table 1 lists reactions that also include the species *M*, which denotes a third-body. In these reactions, the collisions between the third-body and the other molecules provide the energy required for activation and enable the reaction. The third-body can be any atom or molecule present in the system. In such reactions, it is customary to include a collision efficiency factor, η, in the rate expression to account for the difference in the amount of energy provided by collisions involving different third-bodies. The reported η values in Table 1 are given on the basis of argon having the efficiency of 1. In the original work of Kamath et al. [8], the collision efficiency factor values used for the unspecified species, e.g. $H_{2}$ in the seventh reaction in Table 1, is not clearly described. In this work, an efficiency factor of 1 is assumed for these collisions. The effect of η can be taken into consideration by modifying the third-body concentration

(7) $$c_{M}=\overset{N_{T}}{\underset{i=1}{\sum }}\eta_{i}c_{i}$$

which is then used in Eqs. (5), (6) for the third-body reactions. Here $N_{T}$ is the number of species present in the system.

#### 2.2.3 Species mass and energy balances in the reactive system

The species formed in the collapsing cavitation bubble upon dissociation of water vapor molecules at high temperatures may transfer to the liquid phase and result in changes in the bubble composition. Highly oxidative species, such as *OH* radical or $H_{2}O_{2}$, will then rapidly react with the molecules present in the liquid phase, e.g., pollutants in wastewater, to return to a more stable state. Amongst these oxidative species, *OH* radicals are frequently seen as the most important one for advanced oxidation processes due to their strong oxidation potential [38], [39]. Thus, in this work, only the transfer of the hydroxyl radicals are considered as a measure of the performance of different reaction models used. However, the transfer of other species still play an important role in the system, as they result in changes in the bubble composition, and consequently the reaction kinetics and the equilibrium conditions. Therefore, a species mass transfer equation for each species is added to the model:

(8) $${\overset{˙}{N}}_{S}=4\pi R^{2}D_{S}\frac{-\frac{N_{S}}{V_{B}}}{l_{\mathrm{diff},S}}=-\frac{3}{R}D_{S}\frac{N_{S}}{l_{\mathrm{diff},S}}$$

where *S* stands for different species.

The heat of reaction and the transfer of the reaction products through the interface results in two additional terms in the energy balance and Eq. (4) becomes:

(9) $${\overset{˙}{T}}_{B}=\frac{1}{c_{v,B}}\left(4\pi R^{2}\lambda_{B}\frac{T_{L}-T_{B}}{l_{\mathrm{th}}}-4\pi R^{2}\overset{˙}{R}p_{B}+(h_{w}-e_{w}){\overset{˙}{N}}_{w}+\overset{N_{T}}{\underset{i=1}{\sum }}(h_{S}-e_{S}){\overset{˙}{N}}_{S}+\overset{N_{r}}{\underset{i=1}{\sum }}{\mathrm{rV}}_{B}\Delta H_{\mathrm{rxn}}\right)$$

where the last term accounts for the change of energy due to hydroxyl radical mass transfer across the bubble interface. where $N_{r}$ is the number of reactions. The tabulated values for the heat of reaction in Table 1 are used in calculations for the kinetic models. To validate these values, a second approach is followed where the mixture enthalpies obtained through Cantera before and after each reaction step are employed to calculate the overall heat loss or gain of the system due to reactions. Both for the simplified and the full kinetic models, these two approaches are found to yield near identical outcomes, confirming the validity of the tabulated heat of reaction values in the temperature ranges where each reaction is significant. When equilibrium-based approaches, including the peak evaluation method, are employed, the heat of reaction is not commonly taken into consideration in the literature. To be able to compare the predictive power and computational cost of these methods (as they are typically used), the heat of reaction is also neglected in the current study. Then, Eqs. (2), (3) and (8), (9), together with the selected kinetic or equilibrium approach, describe the dynamics of a cavitating bubble whose composition is allowed to change chemically. The expressions and the temperature dependencies required to close the model are presented in the Appendix.

## 3 Numerical procedure

**Fig. 1:**
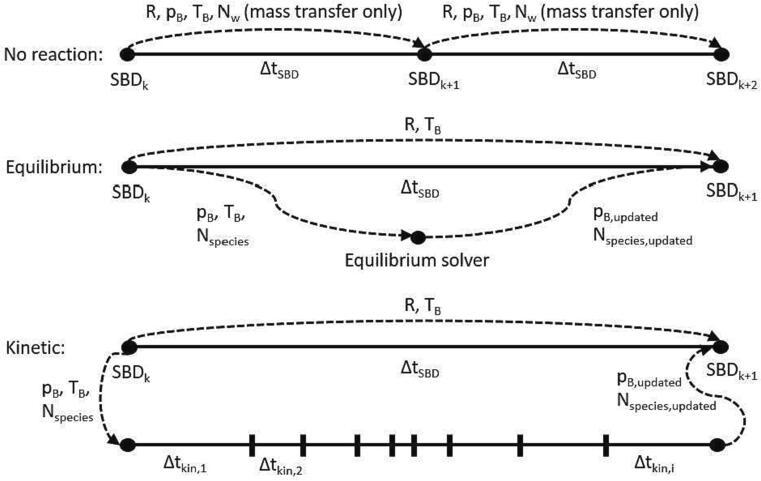
Solution algorithms for no reaction, and reaction with equilibrium and kinetic solver cases. Each circular node in the figure represents solvers, and arrows show the variables passed between the solvers during time-stepping. SBD represents the single bubble dynamics solver, *k* and $\Delta t_{\mathrm{SBD}}$ are the time-step and the time-step size of the SBD solver respectively. $\Delta t_{\mathrm{kin},i}$ shows the time-step size of the kinetic solver.

The model solution is carried out through two consecutive solvers, as shown in Fig. 1 for different reaction cases. At each time step, the change in the chemical composition due to reactions is computed based on the chosen reaction approach. The resulting composition and an updated bubble pressure are then passed to the single bubble dynamics solver, where the bubble radius, temperature and new compositions (considering only physical changes due to mass transfer across the bubble interface) are evaluated following Eqs. (2), (3), (8), (9).

### 3.1 Kinetic/Equilibrium Solver

When the kinetic approach is chosen, a system of ordinary differential equations is solved for the number of molecules of each species based on Eq. (6). A first order forward time discretization scheme is adopted for the integration of the kinetic equations with a time step size that varies with the bubble temperature. This kinetic time step size is often much smaller than the one required for the resolution of the bubble dynamics equations. In this case, the kinetic equations are integrated multiple times until the total integration time in the kinetic solver matches the single bubble solver’s time step size.

For the equilibrium and the peak-evaluation approaches, the equilibrium compositions of the species present in the bubble are determined through the Cantera toolbox that is coupled to the single bubble solver in the same way as the kinetic solver. In the peak-evaluation approach the temperature peaks have to be detected during the solution procedure. However, this is only possible once the bubble temperature from the next time step is known. Therefore, the ’peak-detection’ works retroactively, i.e., first the next time step is solved for and provided that a peak in temperature is detected, the previous time step is re-evaluated by activating the equilibrium solver. Since in a typical simulation only a few temperature peaks are observed, having to evaluate these time steps twice does not affect the overall solver run-time considerably.

### 3.2 Bubble Dynamics Solver

Once the chemical composition of the bubble is evaluated through the kinetic/equilibrium solver, Eqs. (2), (3), (8), (9) are solved simultaneously to yield the bubble radius and temperature, and the new bubble compositions after mass transfer across the interface is considered. Although, in both solvers the bubble composition can change, it must be noted that the chemical changes are accounted for in the kinetic/equilibrium solver, whereas the changes in the bubble dynamics solver are only physical ones. In this solver, the ordinary differential equations are discretized following a first order backwards scheme.

## 4 Results and discussion

The main goal of the present study is to investigate different approaches of modelling the reactions in single bubble dynamics simulations. To this end, the single bubble solver, coupled with the alternative kinetic/equilibrium solvers, described in Sections 2, 3, is applied to a pressure cycle similar to the one investigated by Storey and Szeri [11] for an acoustic cavitation setting. In this configuration, a nucleus, initially filled with argon, is excited with a pressure wave in the form of

(10) $$p_{L}=p_{\mathrm{atm}}-A\sin(2\pi \mathrm{ft})$$

where *A* and *f* are the amplitude and the frequency of the pressure wave, and $p_{\mathrm{atm}}$ is the atmospheric pressure. The single bubble solver has been verified by reproducing Storey and Szeri [11]’s results. Although the experimental validation of the bubble dynamics model is often not straightforward due to lack of information on the precise initial bubble conditions in a typical experiment, Lauterborn and Kurz [40] showed that models built on similar assumptions to the current model are capable of replicating the bubble dynamics observed in experiments. While the objective of the study is to investigate computationally efficient and accurate methods for modelling reactions during hydrodynamic cavitation processes, the acoustic cavitation cycle of Storey and Szeri [11] is considered here since it allows the nature of the cavitation to be changed in a simple and systematic way, by changing the amplitude and frequency of the cycle. In a hydrodynamic cavitation setting, the magnitude and rate of the pressure change that the cavitation bubble experience will also change depending on the design and operation of the cavitation device. The acoustic pressure cycle considered here is therefore used to approximate different qualitative behaviours that will also be present in hydrodynamic cavitation reactors. The conclusions made during this study should therefore be equally relevant when applied to pressure profiles that bubbles experience during hydrodynamic cavitation.

In Section 4.1, a three-dimensional parameter space spanning A,f, and the initial nucleus radius $R_{0}$ is explored to investigate their effects on the bubble dynamics and the radical production behavior over a wide range of bubble collapse conditions. In Section 4.2, a comprehensive comparison between the different kinetics and equilibrium based approaches (and their combinations and variations) is carried out based on their accuracy in estimating the radical production rates and their computational efficiency. In this subsection, the results obtained with the full kinetic model is considered as the basis for the comparison between the radical production predicted by different reaction approaches. For the computational times required on the other hand, the no reaction case is taken as reference, since in this case only the single bubble dynamics equations are solved. As a conclusion, Section 4.2 recommends an optimal approach for chemical reaction modelling that can be employed in detailed reactor simulations, whereas Section 4.3 proposes an algebraic model that estimates the total radical production over an acoustic cycle without solving for the bubble dynamics, which can prove useful for analyses where detailed reactor simulations are not needed.

### 4.1 Reactive bubble dynamics

**Fig. 2:**
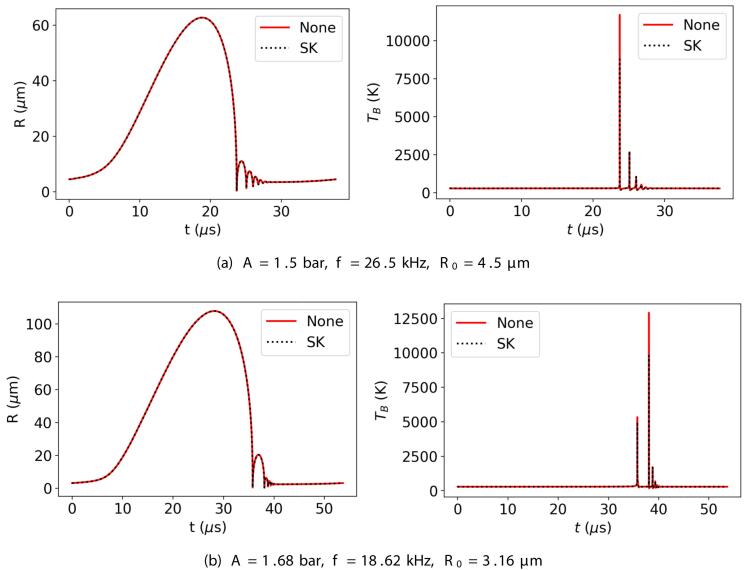
Bubble radius and temperature as a function of time with no reactions and with the simplified kinetic model.

**Fig. 3:**
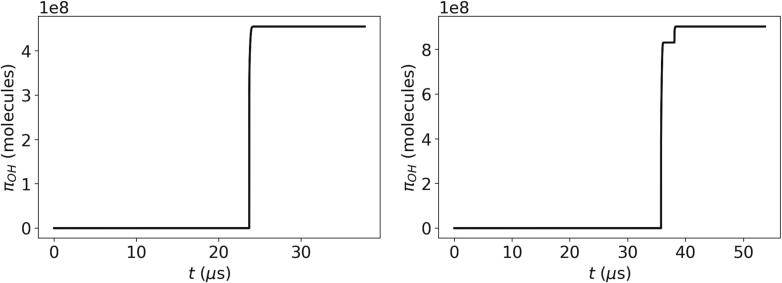
Cumulative amount of radicals transferred to the liquid phase as a function of time for A=1.5 bar, f=26.5 kHz, $R_{0}=4.5$μm (left), and for A=1.68 bar, f=18.62 kHz, $R_{0}=3.16$μm (right) with the simplified kinetic model.

Fig. 2 presents examples of the time evolution of the bubble radius and temperature over one acoustic cycle for two different cases: by considering no chemical reactions in the bubble (None), and by assuming the simplified kinetic model (SK) holds. As can be seen, the bubble radius is visually indistinguishable for the two cases, i.e., the chemical changes in the bubble content have little to no effect on the time evolution of the bubble radius. Although this is shown only for two parameter sets and a single reaction model for the sake of simplicity, this conclusion holds for all the tested points and reaction approaches (the deviations in the simulated bubble radius compared to the no reaction case are typically found to be less than 1%). The bubble temperature, on the other hand, appears to be lower when the chemical reactions are considered, especially at higher temperatures. The difference between the peak $T_{B}$ values obtained for SK and None cases is as large as a few thousand Kelvins. Although not explicitly shown here, this difference is found to disappear when the heat of reaction is not considered in the model. This confirms that the inclusion of heat of reaction is of key importance in determining $T_{B}$ accurately, in accordance with the findings of Dehane et al. [37]. A further observation can be made regarding the time where the highest $T_{B}$ is observed. The two cases presented in Fig. 2 show the two distinct behaviors observed throughout this study: the highest bubble temperature is achieved either at the end of the first collapse of the bubble where the bubble size changes the most drastically (Fig. 2(a)), or at the end of the second collapse where the size change is milder in comparison (Fig. 2(b)). However, in both cases, the radical production is dominated by the first collapse. As can be seen in Fig. 3, the cumulative amount of radicals transferred to the liquid phase, $\pi_{\mathrm{OH}}$, increases drastically in both cases during the first collapse and the contribution of the second collapse (and the subsequent ones) to the production is limited. During the second collapse (and the subsequent ones), the smaller bubble contains much fewer water molecules that can be converted to *OH* radicals, and consequently the amounts produced in these collapses are much less than the first collapse, regardless of whether the maximum bubble temperature is achieved at the end of the first or the second collapse.

**Fig. 4:**
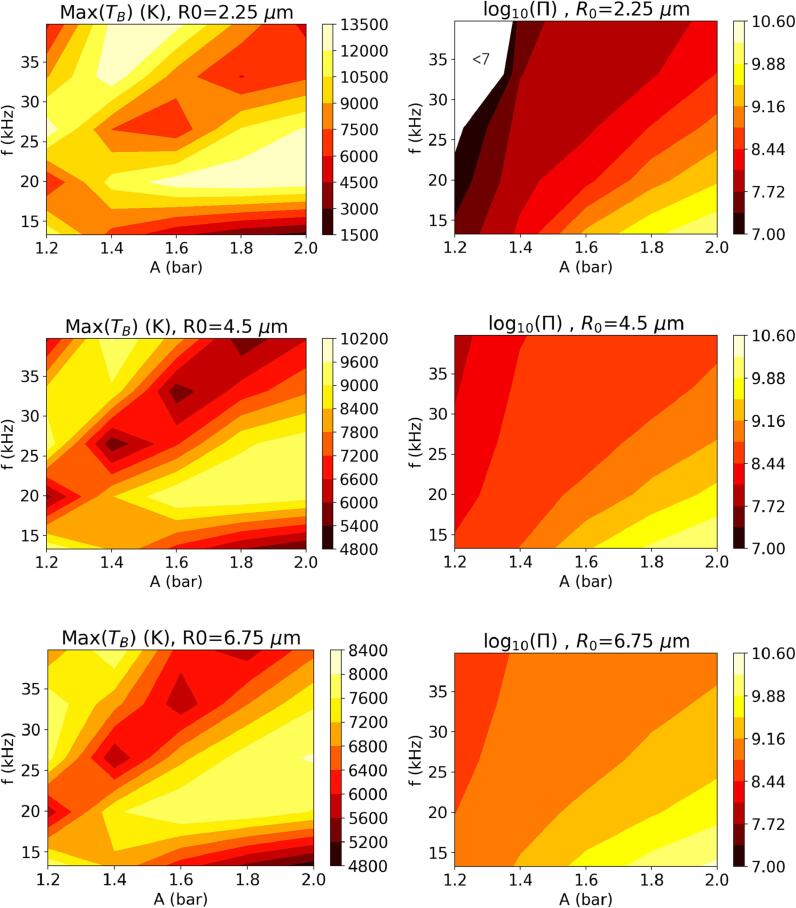
Maximum $T_{B}$ and total radical production over an acoustic cycle, Π(molecules), as a function of *A* and *f* for different initial nucleus sizes.

The cumulative amount of radicals transferred to the liquid phase over one acoustic cycle, Π, i.e., the final value of $\pi_{\mathrm{OH}}$ at the end of the cycle, and the maximum temperature achieved during the cycle are presented in Fig. 4 for the simplified kinetic model for three different values of $R_{0}$ as functions of *A* and *f*. It must be noted that Π here is based on the amount of *OH* radicals transferred to the liquid phase and can be considered a good measure of performance in applications where radical reactions in the liquid phase is required, e.g., wastewater treatment. However, the ultimate performance of the system would depend on the selectivity of the targeted reaction over the competing ones that would scavenge the available radicals. Furthermore, in applications where the targeted reaction may occur in the gas phase, e.g., degradation of volatile pollutants, degradation kinetics within the bubble should also be taken into consideration. Such systems are excluded from the current analysis.

Fig. 4 reveals that, for each initial bubble size considered, two diagonal bands of high temperature collapse exist, with a trough of relatively low collapse temperatures occurring in between in the *A*-*f* parameter space. A closer inspection of the time evolution of $T_{B}$ shows that this behavior coincides with the earlier observation presented through Fig. 2: The maximum $T_{B}$ is reached at the end of the first collapse of the bubble in the first high temperature region (at smaller amplitudes) until the trough ends, and at the end of the second collapse after the trough. Higher drive frequencies typically appear to require larger amplitudes to achieve the same collapse temperature, resulting in the diagonal band behavior. The collapse temperature increases substantially with decreasing initial bubble size.

Viewing the total *OH* radical production on the right-hand side of Fig. 4, for the effect of *A* and *f* on Π, clear conclusions can be drawn within the investigated parameter space: larger amplitudes and lower frequencies result in more radical production. As *A* increases, the driving force behind the phase change of water molecules becomes stronger, resulting in higher $H_{2}O$ content in the bubble, which in turn reacts to yield radicals under extreme cavitation conditions. The lower values of *f* can be interpreted as bubbles being exposed to these stronger driving forces for a longer duration, which increases the radical production in a similar fashion. These observations are in line with the literature, e.g., Pandit et al. [34]’s findings. Furthermore, it is observed that increasing the initial nuclei size increases the total *OH* radical production. Specifically for $R_{0}=2.25\mu$m, Π decreases very steeply in the low *A* and high *f* limit of the parameter space (as low as $\log_{10}(\Pi )\approx 2.3$) indicating that cavitation in this region is not intense enough to produce a significant amount of radicals. It can be noted that in this region the maximum collapse temperature is below approximately 3000 K, at which significant concentrations of *OH* radicals are not likely to be produced at equilibrium. Increasing the duration the bubble is exposed to $p_{L}⩽p_{\mathrm{vap}}$ and the magnitude of the driving force, by lowering *f* and increasing *A* respectively, result in up to a million times increase in Π and brings estimated Π values for $R_{0}=2.25\mu$m within an order of magnitude of the estimates obtained for the other two initial nucleus sizes.

**Fig. 5:**
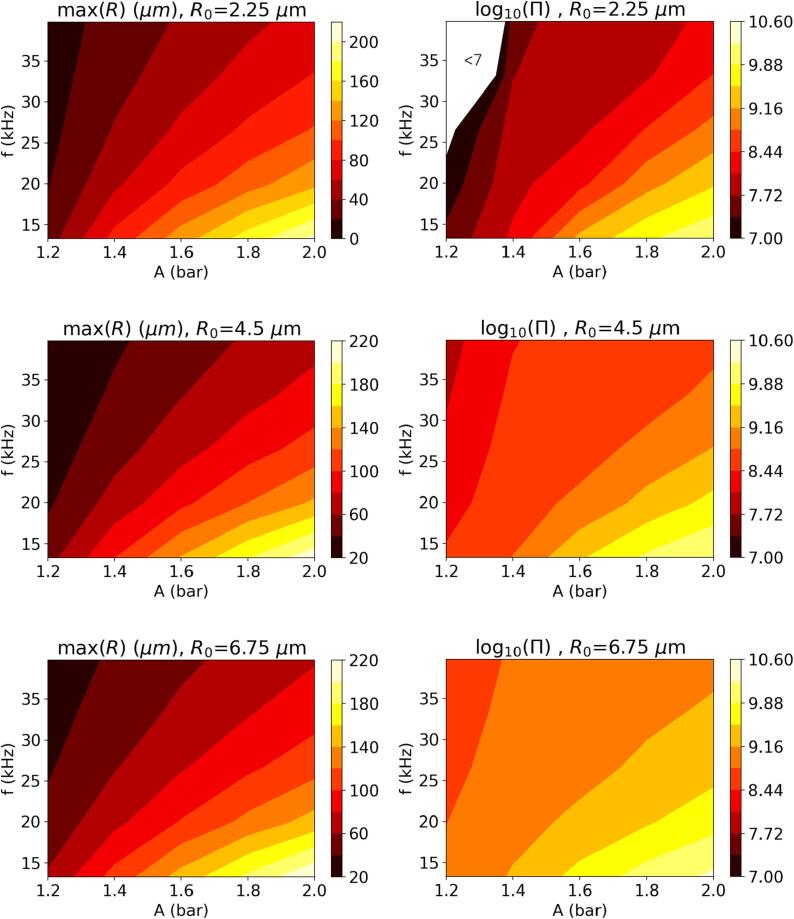
Maximum *R* and total radical production over an acoustic cycle, Π(molecules), as a function of *A* and *f* for different initial nucleus sizes.

Comparison between the maximum $T_{B}$ and Π behaviors given in Fig. 4 clearly indicates that higher maximum bubble temperatures do not necessarily yield larger Π. The correlation between the two can be quantified via the Pearson correlation coefficient defined as

(11) $$\rho_{\mathrm{XY}}=\frac{\mathrm{cov}(X,Y)}{\sigma_{X}\sigma_{Y}}$$

where *X* and *Y* are the two variables of interest, cov(X,Y) their covariance, and $\sigma_{X}$ and $\sigma_{Y}$ their standard deviations. Values of $\rho_{\mathrm{XY}}=1$ and $\rho_{\mathrm{XY}}=-1$ indicate a perfect positive and a perfect negative correlation, respectively, whereas $\rho_{\mathrm{XY}}=0$ implies that there is no correlation between the two variables. For $R_{0}=[2.25,4.5,6.75]$ μm, $\rho_{\mathrm{XY}}=[0.5019,0.1640,0.3001]$ when $T_{B}$ and $\log_{10}(\Pi )$ are considered, confirming that there is no strong correlations between these two quantities. Extending this analysis to investigate possible correlations between $\log_{10}(\Pi )$ and the maximum *R* and $p_{B}$, as well as between $\log_{10}(\Pi )$ and $T_{B},R$ and $p_{B}$ values at the end of the first collapse (denoted with $T_{B,1},R_{1}$, and $p_{B,1}$ hereafter) yields the $\rho_{\mathrm{XY}}$ values presented in Table 2. It must be noted that $\max(T_{B})$ and $T_{B,1}$ (likewise $\max(p_{B})$ and $p_{B,1}$) may coincide, yet max(R) and $R_{1}$ are different as the bubble size reaches a local minimum at the end of the collapse. The results indicate that the largest bubble size achieved in an acoustic cycle is a good indicator of the total amount of radicals produced in the cycle, especially for $R_{0}⩾4.5\mu$m, where an almost perfect positive correlation is observed with the values $\rho_{\mathrm{XY}}=0.9841$ and $\rho_{\mathrm{XY}}=0.9808$ for $R_{0}=4.5\mu$m and $R_{0}=6.75\mu$m, respectively. For $R_{0}=2.25\mu$m, on the other hand, $\rho_{\mathrm{XY}}=0.7615$, which still shows a strong correlation despite being weaker than the other two bubble sizes. As can be seen in Fig. 5, the maximum bubble size and Π correlate well except the small *A* and high *f* limit for $R_{0}=2.25\mu$m, where cavitation is not intense enough to produce significant amounts of radicals (as discussed for Fig. 4). Neglecting this region (specifically two data points: f=39.75 kHz, A=1.2 bar andf=33.125 kHz, A=1.2 bar) in the correlation analysis results in $\rho_{\mathrm{XY}}=0.9792$. The new value of $\rho_{\mathrm{XY}}$ shows that the largest bubble size is a good indicator for Π for $R_{0}=2.25\mu$m as well, provided that the cavitation is intense enough to reach a certain threshold temperature, above which significant concentrations of *OH* radicals are produced. Although the other variables investigated in Table 2 exhibit moderate correlation with $\log_{10}(\Pi )$, none of them yields $\left|\rho_{\mathrm{XY}}\right|$ close to 1, as the maximum bubble radius does.

**Table 2:** Correlation coefficient between $\log_{10}(\Pi )$ and $T_{B},R$, and $p_{B}$ achieved at the end of the first collapse, and their maximum values.

X,Y	$R_{0}=2.25\mu$m	$R_{0}=4.5\mu$m	$R_{0}=6.75\mu$m
$\max(T_{B})(K),\log_{10}(\Pi )$	0.5019	0.1640	0.3001
$\max(R)(\mu m),\log_{10}(\Pi )$	0.7615	0.9841	0.9808
$\max(p_{B})(\mathrm{Pa}),\log_{10}(\Pi )$	0.5839	0.4960	0.5693
$T_{B,1}(K),\log_{10}(\Pi )$	0.0705	-0.5986	-0.5623
$R_{1}(\mu m),\log_{10}(\Pi )$	0.2979	0.6743	0.4754
$p_{B,1}(\mathrm{Pa}),\log_{10}(\Pi )$	0.3598	0.2771	0.4580

The trends observed in this section imply that, for cavitation intensification applications relying on *OH* radical production, maximizing the collapse temperature (which may be seen as an indicator of the collapse intensity) of individual bubbles does not guarantee improved process performance, although a certain level of collapse intensity is required for the onset of *OH* radical production. Instead, when the collapse intensity is sufficient, maximizing the water vapor available for conversion to *OH* radicals (indicated by, e.g., maxR) is more important. This implies that for optimal operation of a hydrodynamic cavitation device an optimal collapse intensity will exist which maximizes the radical production per unit energy input required to produce cavitation. Also, this has important implications for certain hydrodynamic cavitation applications, where long-term operation with intense collapse might lead to excessive erosion of the device due to shockwaves produced during collapse, but possibly without an improvement in process performance.

### 4.2 Comparison between different reaction modelling approaches

A central composite design is constructed to effectively evaluate the impact of the different kinetic and equilibrium based approaches on the system while keeping the number of runs relatively low. Table 3 presents the design points used in the analysis, where the axial points were chosen to be on the outer edges of the ranges investigated in Section 4.1. The solver is then run for each of these points by considering the different kinetic/equilibrium approaches described in Section 2.2, as well as their combinations and variations, i.e.,

• no reaction (None),
• full kinetic model (FK),
• simplified kinetic model (SK),
• peak evaluation approach (Peak),
• peak evaluation followed by full kinetic model (PF),
• peak evaluation followed by simplified kinetic model (PS),
• equilibrium evaluation when $T_{B}$ is greater than a temperature threshold $T_{c}$ (TC).

**Table 3:** Points generated through a central composite design.

Design #	*A* (bar)	*f* (kHz)	$R_{0}$ (μm)
0	1.5	26.50	4.50
1	1.8	26.50	4.50
2	1.2	26.50	4.50
3	1.5	39.75	4.50
4	1.5	13.25	4.50
5	1.5	26.50	6.75
6	1.5	26.50	2.25
7	1.68	34.38	5.84
8	1.32	34.38	5.84
9	1.68	18.62	5.84
10	1.32	18.62	5.84
11	1.68	34.38	3.16
12	1.32	34.38	3.16
13	1.68	18.62	3.16
14	1.32	18.62	3.16

**Fig. 6:**
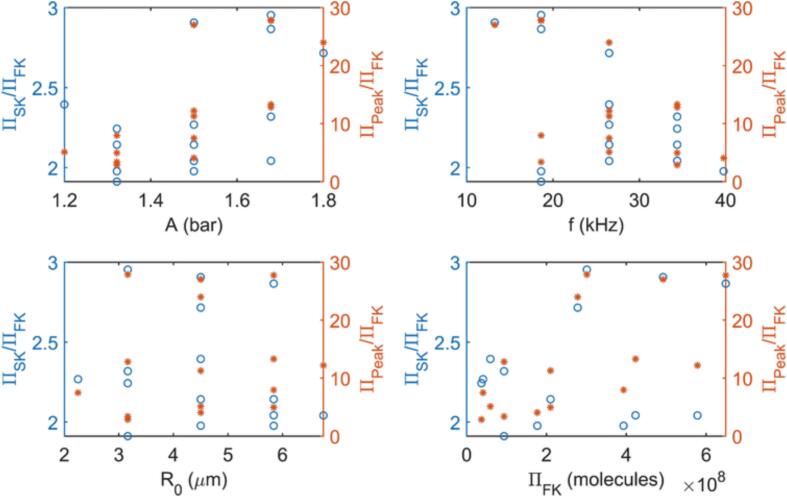
Ratio of the total radical production predicted by simplified kinetic and peak evaluation approaches to the full kinetic model’s outcome as a function of $A,f,R_{0}$ and $\Pi_{\mathrm{FK}}$.

Fig. 6 presents the ratio of Π values predicted by the simplified kinetic and the peak evaluation approaches to the total production estimated by the full kinetic model, $\Pi_{\mathrm{FK}}$, at the points given in Table 3. Both approaches predict larger amounts of radicals produced than the full kinetic model regardless of the values of A,f, and $R_{0}$, and $\Pi_{\mathrm{FK}}$. The simplified kinetic model yields on average $\Pi_{\mathrm{SK}}\approx 2.3\Pi_{\mathrm{FK}}$, with a maximum of $\Pi_{\mathrm{SK}}\approx 2.9\Pi_{\mathrm{FK}}$ and a minimum of $\Pi_{\mathrm{SK}}\approx 1.9\Pi_{\mathrm{FK}}$. Although $\Pi_{\mathrm{SK}}/\Pi_{\mathrm{FK}}$ exhibits a relatively small deviation, with a coefficient of variation of 15.6%, it can be seen that SK over-estimates Π by a larger margin as *A* increases and *f* decreases. Both large amplitudes and low frequencies enhance the radical production as discussed in Section 4.1, indicating that the accuracy of SK estimations is lower when the total radical production is higher. On the other hand, $\Pi_{\mathrm{SK}}/\Pi_{\mathrm{FK}}$ shows no clear deviation trends against the changes in $R_{0}$. The conclusions regarding the trends also hold for $\Pi_{\mathrm{Peak}}/\Pi_{\mathrm{FK}}$, yet it must be noted that the discrepancy between the predictions of the peak-evaluation method and $\Pi_{\mathrm{FK}}$ is far more significant. The total production in this case can be as large as $\Pi_{\mathrm{Peak}}\approx 30\Pi_{\mathrm{FK}}$, with a minimum of $\Pi_{\mathrm{Peak}}\approx 2.9\Pi_{\mathrm{FK}}$. It is also seen that the deviation in $\Pi_{\mathrm{Peak}}/\Pi_{\mathrm{FK}}$ is considerably higher in all subplots in Fig. 6. The mean value of $\Pi_{\mathrm{Peak}}/\Pi_{\mathrm{FK}}$ is calculated as 12.8, with a coefficient of variation of 72.8% compared to a value of 15.6% for the SK case. This indicates that the peak evaluation method may yield less reliable predictions of Π, both quantitatively and when evaluating qualitative trends with changing collapse conditions. The fact that heat of reaction is neglected in the peak evaluation method is found to play a minor role in explaining the discrepancy between its estimations and those of the kinetic models, as $\Pi_{\mathrm{Peak}}/\Pi_{\mathrm{FK}}$ is still as large as 8.90 on average when both methods do not consider the heat of reaction.

**Fig. 7:**
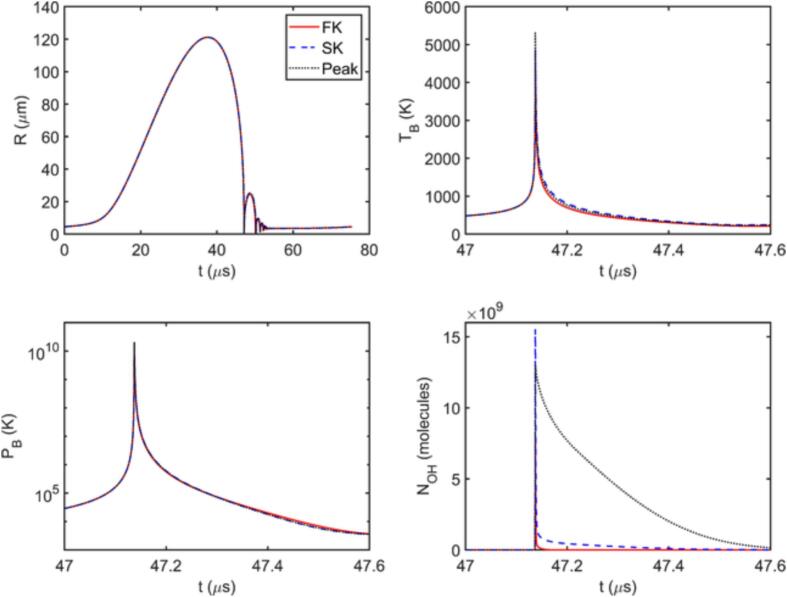
Time evolution of *R* over one acoustic cycle, and $T_{B},p_{B}$, and number of *OH* molecules in the bubble during the first collapse for A=1.5 bar, f=13.25 kHz and $R_{0}=4.5\mu$m.

**Fig. 8:**
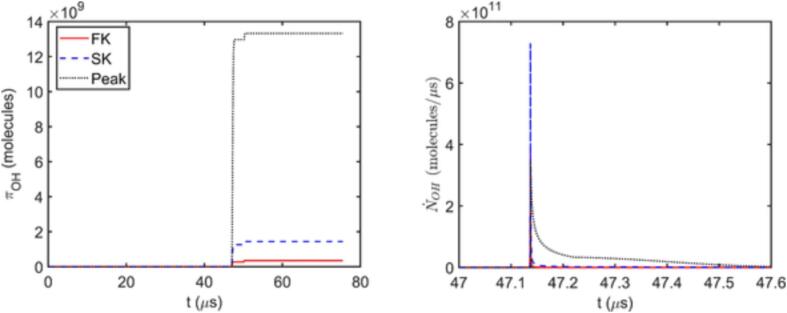
Time evolution of $\pi_{\mathrm{OH}}$ over one acoustic cycle and the rate of *OH* transfer across the interface ${\overset{˙}{N}}_{\mathrm{OH}}$ during the collapse for A=1.5 bar, f=13.25 kHz and $R_{0}=4.5\mu$m.

The reason behind the differences in Π values predicted by SK, PK, and Peak approaches can be revealed by analyzing the time evolution of number of *OH* molecules in the bubble and *OH* transfer rate across the bubble interface, ${\overset{˙}{N}}_{\mathrm{OH}}$. Fig. 7 shows that $N_{\mathrm{OH}}$ behavior predicted by each approach is different, even though their estimations for $R,T_{B}$, and $p_{B}$ are nearly identical except for the peak bubble temperature. In all three cases, $N_{\mathrm{OH}}$ increases very steeply following the sudden increase in $p_{B}$ and $T_{B}$ during the collapse. The produced radicals can then either be transferred to the liquid phase or be depleted in further reactions within the bubble. Considering Fig. 7, Fig. 8 together further elaborates the differences in predictions of the three approaches. The full kinetic model estimates a very sharp drop in $N_{\mathrm{OH}}$ right after its peak, implying rapid radical consumption in the bubble, which limits the amount of radicals transferred across the interface. The simplified kinetic model appears to approximate this behavior relatively well, except that it yields larger $N_{\mathrm{OH}}$ at the peak and a slightly less dramatic drop followed by a gradual decrease in $N_{\mathrm{OH}}$, both of which contribute to the larger Π predictions. This is likely because the simplified kinetic model neglects several reaction pathways where the produced OH radicals can be converted further to alternative species, such as $H_{2}O_{2}$ or ${\mathrm{HO}}_{2}$ radicals. The peak evaluation method predicts lower maximum $N_{\mathrm{OH}}$ than SK, yet it fails to capture the sudden drop as it does not consider any possible reactions occurring after the peak. Since the over prediction of the produced radicals is generally much greater for the peak evaluation approach than for the simplified kinetics, it is evident that the reactions converting *OH* radicals back to water in the reactions included in the simplified kinetics is of high importance in the time period following the peak temperature. Neglecting these reactions eventually leads to transfer of all the produced radicals to the liquid phase and consequently to considerably over predicted Π values with the peak evaluation approach.

In addition to the comparison of the Π estimations, the computational run times required for different approaches should also be taken into consideration. For acoustic cavitation studies of a single bubble, even the most computationally demanding full kinetic model can be solved in reasonable computational times. However, employing the framework presented here for a hydrodynamic cavitation setting, where a large number of bubbles following different trajectories are likely to cavitate simultaneously and interact with each other, brings out several challenges, one of which is the computational cost of modelling the reactions. Thus, to render the extension of the single bubble simulations to hydrodynamic cavitation studies possible, a reaction modelling method that is capable of yielding reasonable radical production rates without requiring immense computational resources should be employed. None of the approaches discussed so far satisfies these two requirements simultaneously. On average, the full kinetic model requires roughly 16 times longer simulation times compared to the no reaction case. The simplified kinetic model approximately triples the computational time required for the no reaction case, and over estimates the total radical production predicted with the full model by a factor of 2.3 on average. The peak evaluation approach is capable of resulting in similar run times to the no reaction case, yet it may yield an order of magnitude larger Π.

Therefore, the analysis is extended by including further variations (and combinations) of the approaches discussed so far. In the first category, peak evaluation is followed by solving the full kinetic model (PF) or simplified kinetic model (PS), where the kinetic equations are only solved after the equilibrium composition is evaluated at peak temperatures. These options are suggested based on the finding that the peak evaluation approach over predicts the radical production due to neglecting the reactions reverting OH radicals to water following the peak temperature. Therefore, by solving the kinetic equations only after evaluating the equilibrium at the peak temperature, the computational cost could be reduced (compared to the standard kinetic approaches), while improving the accuracy (compared to the standard peak evaluation approach). As a second category of alternative options, the equilibrium approach is investigated, where the equilibrium compositions are evaluated continuously provided that $T_{B}$ is larger than a certain threshold $T_{c}$. This is suggested based on the argument that, above a given temperature, the reaction kinetics will be fast enough that the composition will always approach equilibrium. To analyze the predictive power and the computational performance of all the proposed approaches two criteria are created: the absolute value of the percent difference between the Π predictions of the investigated approach and the full kinetic model, $C_{1}$, and the percent increase in the computational time relative to the no reaction case, $C_{2}$. The sought-after approach should ideally yield as low as possible $C_{1}$ and $C_{2}$ values.

**Fig. 9:**
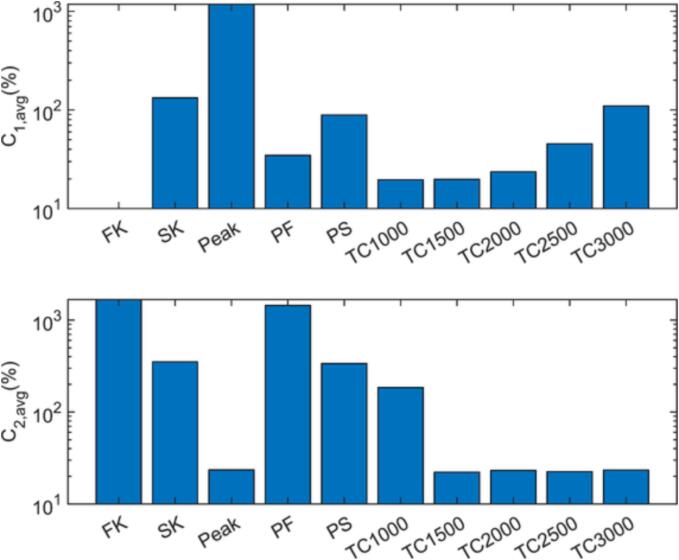
$C_{1}$ and $C_{2}$ averaged over the 15 design points in Table 3 for different reac.tion approaches.

Fig. 9 presents the averaged $C_{1}$ and $C_{2}$ values over the design points given in Table 3 for the different reaction modelling approaches studied in this work. The results clearly show the trade-off between the accuracy of the predicted radical production rates (through $C_{1}$) and the computational costs (through $C_{2}$) for the FK, SK, and Peak cases, supporting the earlier observations. The newly introduced PF and PS cases, that combine the peak evaluation and kinetic approaches, succeed in bringing the production estimates closer to $\Pi_{\mathrm{FK}}$ and reduce $C_{1}$ considerably in comparison to the peak-evaluation method. However, both alternatives fail to decrease the computational costs considerably as evident from large values of $C_{2}$. As the kinetic equations require the smallest time step sizes at higher bubble temperatures, i.e., around the peak temperatures, enabling the kinetics after the peak evaluation means that these equations are still solved with very fine time-stepping in the PF and PS approaches. Consequently, the computational costs of PF and PS still remain high despite minor improvements in $C_{2}$ values in comparison with their respective counterparts FK and SK.

For the equilibrium approaches, it is seen that the computational costs decrease considerably for $T_{c}>1000K$ and $C_{2}$ approaches that of the peak evaluation approach. This improvement in the computational performance is well-expected as increasing $T_{c}$ decreases the number of evaluations of the equilibrium compositions. The predictive power of the model on the other hand, is expected to be weak for very low $T_{c}$, as at lower temperatures it is not likely that the chemical reactions reach equilibrium, and for very high $T_{c}$, since the production would not be evaluated at $T_{B}$ values that can result in significant changes in the bubble composition. In other words, an optimal $T_{c}$ value or range that yields low $C_{1}$ is likely to exist. As can be seen, both for $T_{c}=1000K$ and $T_{c}=1500K$, the equilibrium evaluations predict Π values that are very close to $\Pi_{\mathrm{FK}}$ (19.6% and 19.8% off on average) even though the heat of reaction is neglected in the equilibrium approaches, and $C_{1}$ only increases starting with $T_{c}=2000K$. At these higher temperatures, the radical production is generally over predicted, since reactions during portions of the bubble collapse significantly contributing to the radical consumption within the bubble is discarded due to the bubble being below the threshold temperature. Despite the assumption of reaching equilibrium being less likely at lower temperatures, the high accuracy at TC1000 is likely due to the equilibrium *OH* radical concentration being negligible at low temperatures. Consequently, employing the equilibrium assumption at lower temperatures, where it is potentially unrealistic, has little effect on the results. However, using a very low threshold temperature greatly increases the number of time steps where the equilibrium compositions has to be estimated, substantially increasing the computational cost. In conclusion, for the evaluated system, employing the equilibrium approach with the threshold in the vicinity of $T_{c}=1500K$ is found to be the best option, which could potentially be used in studies considering a large number of cavitating bubbles simultaneously, e.g., in a hydrodynamic cavitation device.

### 4.3 Algebraic model for estimating radical production

As the time evolution of bubble radius is not affected by the chemical reaction modelling (as discussed for Fig. 2), the strong correlation between max(R) and $\log_{10}(\Pi )$ discussed in Section 4.1 implies that the extent of radical production (in terms of order of magnitude) can be estimated without directly solving the chemical reactions in simulations for the cavitation of a single isolated bubble, as long as the bubble temperature reaches values that are large enough to initiate the chain of reactions resulting in *OH* production. This conclusion can be further extended to single bubble cavitation experiments: the measurement of the largest bubble size in an experiment may be sufficient to qualitatively assess the conditions for maximum *OH* production without requiring the *OH* concentration to be measured. However, in either case, a less expensive simulation or a less tedious experiment should be run to determine max(R). To avoid this, Π must be estimated from simulation/experimental inputs, such as *A* and *f*. As discussed in Section 4.1, the amount of water vapor transferred to the bubble during cavitation bubble growth is a good indicator of the amount of radicals produced, given that the cavitation collapse is sufficiently intense. An indicator of the expected extent of the water vapor transfer as a function of *A* and *f* can be created by manipulating Eq. (10), which gives the sinusoidal pressure wave applied to the system. Over one pressure cycle, the liquid pressure given by Eq. (10) intersects with $p_{\mathrm{vap}}$ twice, respectively marking the time $p_{L}$ drops below $p_{\mathrm{vap}},t_{0}$, and the time it increases above $p_{\mathrm{vap}}$ once again, $t_{f}$. The times $t_{0}$ and $t_{f}$ are given by

(12) $$t_{0}=\sin^{-1}\left(\frac{p_{\mathrm{atm}}-p_{\mathrm{vap}}}{A}\right)\frac{1}{2\pi f}$$

and

(13) $$t_{f}=\left[\pi -\sin^{-1}\left(\frac{p_{\mathrm{atm}}-p_{\mathrm{vap}}}{A}\right)\right]\frac{1}{2\pi f}$$

However, the duration the bubble is exposed to $p_{L}⩽p_{\mathrm{vap}}$ is not sufficient to estimate the extent of the mass transfer and the consequent bubble growth by itself, as it does not carry information about the instantaneous strength of the driving force. To include the effect of both the strength and the duration, the area under the $p_{L}$-curve when $p_{L}⩽p_{\mathrm{vap}}$ is taken as the indicator:

(14) $$\begin{array}{c} A_{p}=\int_{t_{0}}^{t_{f}}\left[p_{\mathrm{vap}}-\left(p_{\mathrm{atm}}-A\sin(2\pi \mathrm{ft})\right)\right]\mathrm{dt}\\ ={\left(\left[-\frac{A}{2\pi f}\cos(2\pi \mathrm{ft})+\left(p_{\mathrm{vap}}-p_{\mathrm{atm}}\right)t\right]\right|}_{t=t_{0}}^{t=t_{f}} \end{array}$$

**Fig. 10:**
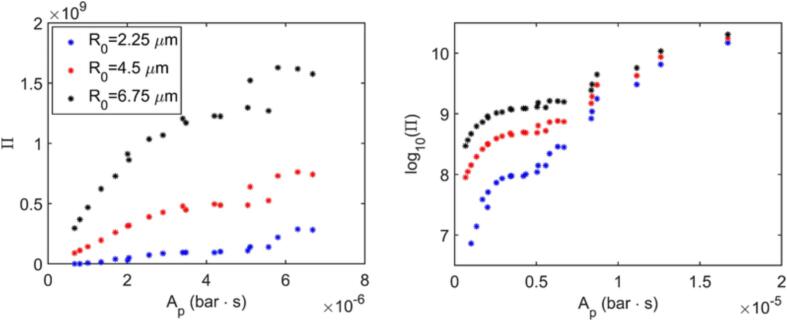
Cumulative *OH* production as a function of $A_{p}$ for different initial bubble radii.

Fig. 10 presents the Π values given in Fig. 5 as a function of $A_{p}$ for different initial bubble radii, where the simplified kinetic model is used. Fig. 10 reveals two distinct trends. At lower values of $A_{p}$, roughly for $A_{p}<8\times 10^{-6}$ bar·s,Π first increases linearly with $A_{p}$ for all $R_{0}$ until $A_{p}\approx 5\times 10^{-6}$ bar·s, after which the increase in Π starts deviating from the linear trend, e.g., the increase diminishes for $R_{0}=6.75\mu m$. A linear trend can be expected, since the water transfer to the bubble would be proportional to $A_{p}$. The deviation from linear behaviour at larger $A_{p}$ is likely due to the onset of mass transfer limitations that restrict evaporation when further increasing $A_{p}$. For even larger values of $A_{p}$, the increase in Π becomes near exponential instead, indicating that a change in the radical production characteristics is likely. The transition between the two trends also coincides with the earlier observation on the peak bubble temperature: for $A_{p}<8\times 10^{-6}$ bar·s, it is found that the highest $T_{B}$ is achieved during the first collapse of the bubble, and for $A_{p}>8\times 10^{-6}$ bar·s in the second one.

**Fig. 11:**
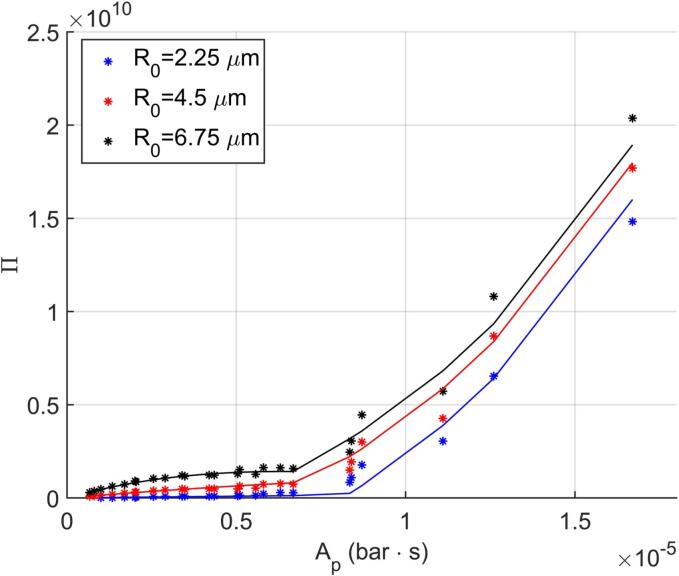
Cumulative *OH* production estimated by the reactive bubble dynamics model with simplified kinetic model (stars) and by Eq. (15) (solid lines) as a function of $A_{p}$ and $R_{0}$.

The two distinct production trends seen in Fig. 10 can be approximated with a piecewise function in the form of

(15) $$\Pi =\left\{\begin{array}{c} \beta_{1}\frac{10^{\beta_{2}A_{p}}}{1+10^{\beta_{2}A_{p}}}A_{p},\;A_{p}<8\times 10^{-6}\;\mathrm{bar}\cdot s\\ \beta_{3}10^{\beta_{4}A_{p}}+\beta_{5},\;A_{p}⩾8\times 10^{-6}\;\mathrm{bar}\cdot s \end{array}\right)$$

where the fitting parameters $\beta_{1}-\beta_{5}$ are allowed to be a function of $R_{0}$. Fig. 11 presents the best-fitting model over the data with ${\left(\rho_{\mathrm{XY}}\right)}^{2}=0.986$ obtained with the parameters

(16) $$\begin{array}{cc} & \beta_{1}=3.31e12\;R_{0}^{3}\\ & \beta_{2}=-281.8\;R_{0}^{3}\\ & \beta_{3}=3.76e9\\ & \beta_{4}=4.98e4\\ & d\beta_{5}=-1.25e10\;R_{0}^{-1/3} \end{array}$$

where $R_{0}$ is in μm. The dependencies of $\beta_{1}$ and $\beta_{2}$ on $R_{0}^{3}$ indicates that the first trend is governed by $A_{p}$ and the initial volume of the nuclei, whereas for the second part of the piecewise function, no such relation is found. The resulting fit is capable of accurately estimating the Π values when $A_{p}<8\times 10^{-6}$ bar·s. Above $A_{p}=8\times 10^{-6}$ bar·s, on the other hand, the predictive power of the fitted model is lower in comparison due to the larger variability in the data, yet the model satisfactorily captures the trends with both $A_{p}$ and $R_{0}$.

**Fig. 12:**
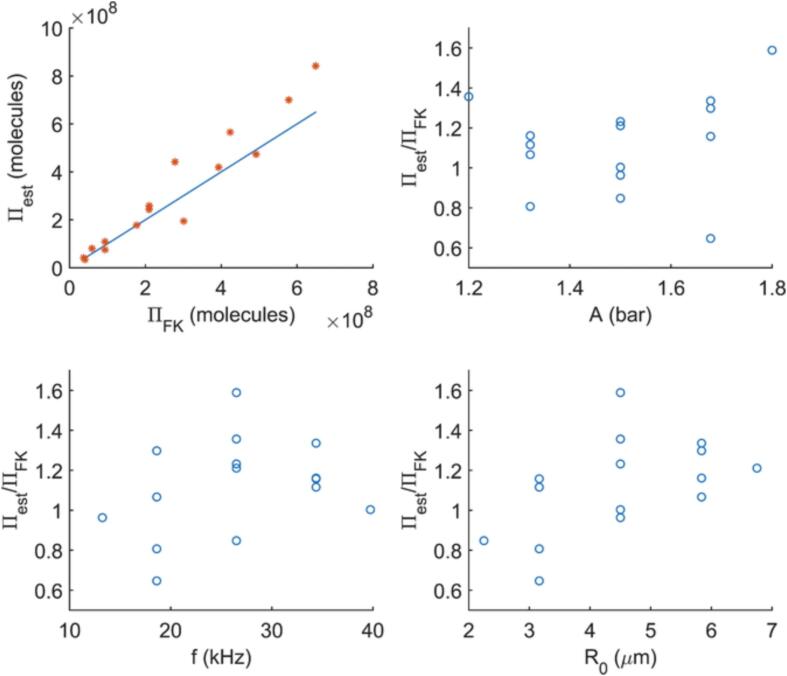
Parity plot between $\Pi_{\mathrm{FK}}$ obtained through the bubble dynamics model with the full kinetics and $\Pi_{\mathrm{est}}$ estimated by Eq. (17), together with $\Pi_{\mathrm{est}}/\Pi_{\mathrm{FK}}$ as a function of A,f and $R_{0}$.

Although the expression is originally fitted by using the results of the simplified kinetic model, by utilizing the conclusion drawn from Fig. 6, i.e., $\Pi_{\mathrm{SK}}/\Pi_{\mathrm{FK}}\approx 2.3$, a relation for $\Pi_{\mathrm{FK}}$ can be written as

(17) $$\Pi_{\mathrm{FK}}=\left\{\begin{array}{c} \frac{1}{2.3}\left(\beta_{1}\frac{10^{\beta_{2}A_{p}}}{1+10^{\beta_{2}A_{p}}}A_{p}\right),A_{p}<8\times 10^{-6}\;\mathrm{bar}\cdot s\\ \frac{1}{2.3}\left(\beta_{3}10^{\beta_{4}A_{p}}+\beta_{5}\right),A_{p}⩾8\times 10^{-6}\;\mathrm{bar}\cdot s \end{array}\right)$$

with $\beta_{1}$ through $\beta_{5}$ being subject to Eq. (16). This expression is then tested by comparing the simulation results of the bubble dynamics model with full kinetics, $\Pi_{\mathrm{FK}}$, and the outcome of Eq. (17), $\Pi_{\mathrm{est}}$, at the design points presented in Table 3. Notice that these points are not amongst the ones employed in the original fitting procedure. Fig. 12 presents the parity plot $\Pi_{\mathrm{FK}}$ and $\Pi_{\mathrm{est}}$ together with their ratio as a function of A,f, and $R_{0}$. As can be seen, the proposed expression is able to predict the simulated $\Pi_{\mathrm{FK}}$ reasonably well, although the test points (Table 3) are not amongst the ones used for the fitting and the original fitting is done with the simplified kinetic model. This indicates that the algebraic expression given in Eq. (17) can be employed without requiring the bubble dynamics to be resolved, to estimate the resulting value of Π, once the liquid pressure history is known. For acoustic cavitation, $A_{p}$ can be determined through Eq. (14) by substituting the amplitude and the frequency of the pressure wave in, whereas for the hydrodynamic cavitation $A_{p}$ should be estimated through flow calculations. When the resolution of the local instantaneous values of *OH* concentration is not required, e.g., in simplified process models involving hydrodynamic- or acoustic-cavitation assisted reactors, the expression proposed in Eq. (17) can prove useful in obtaining reasonable estimates for Π, while for detailed reactor simulations an equilibrium based reaction modelling approach combined with the bubble dynamics can be employed as argued in Section 4.2.

## 5 Summary and conclusions

To investigate the utilization of cavitation to intensify a liquid phase reaction due to the production of hydroxyl radicals, the dynamics and the chemical composition of cavitating bubbles are simulated over a range of cavitation conditions. Different reaction modelling approaches, based on either kinetic or equilibrium principles, are employed to examine the time dependent behavior of bubble radius, temperature, chemical composition and radical transfer to the liquid phase. The results indicate that the choice of chemical reaction model, regardless of whether it is based on kinetics or equilibrium, has little to no impact on the time evolution of bubble radius. However, the inclusion of the heat of reaction in kinetic models is essential for accurate estimation of the bubble temperature as it potentially results in a few thousand cooler peak bubble temperatures. The implications of the different reaction modelling approaches on the estimated radical production rates and the cumulative radical production over a pressure cycle are found to be immense. Furthermore, the cumulative radical production correlates exceptionally well with the maximum bubble size achieved during the cycle, with an average Pearson correlation factor of 0.98. No strong correlations are found between the production and the other variables, such as the bubble temperature and pressure, except for the fact that the bubble temperature must reach values large enough to facilitate the water vapor dissociation reactions.

Amongst the tested approaches, a simplified kinetic model considering 4 reactions is found to overestimate the total production by an average factor of 2.3 in comparison to a full kinetic model with (19 reactions) with three times as long simulation times than a case without modelling reactions. The method of evaluating the equilibrium composition only once and assuming all the produced radicals are transported to the liquid phase yields less reliable production estimates due to neglecting the reactions converting *OH* radicals back to water following the peak temperature. However, simulation times are comparable to the no reaction case. An equilibrium approach, where the chemical changes are allowed to occur based on minimization of Gibbs free energy only when bubble temperature is larger than 1500K, can overcome the challenges of both these approaches. It is found to yield very similar radical production estimates compared to the full kinetic model (19.8% average deviation) while requiring an order of magnitude faster computational times (only 22.2% more than the no reaction case). Thus, this approach carries the best potential amongst the evaluated options to be employed in the extension of reactive single bubble models to larger simulation frameworks, e.g., in hydrodynamic cavitation studies, allowing accurate simulations while minimizing the computational cost of solving the trajectories of a larger number of cavitating bubbles.

For modelling efforts that do not require local instantaneous resolution of the radical concentration in the reactor, an algebraic model that successfully replicates the outcome of the reactive bubble dynamics simulations with the full kinetic model is proposed. The expression employs the initial nucleus radius and the time integral of the liquid pressure (when its under vapor pressure) as independent variables, which is taken as an indicator of the overall extent of evaporation during cavitation bubble growth. Both parameters can be estimated/determined without running simulations or experiments for acoustic cavitation applications once the amplitude and the frequency of the pressure wave is set. For hydrodynamic cavitation systems, CFD simulations can be performed to estimate the pressure history of the bubble and calculate the input to the algebraic model. This opens the way for the estimation of the cumulative radical production without resolving the bubble dynamics and chemistry, in models where the impact of the local instantaneous radical concentration values may not be neglected.

Furthermore, this observation paves the way for closure modelling of radical production in CFD models of hydrodynamic reactors. This would allow reasonable estimates of radical production without the need for detailed resolution of the bubble collapse dynamics, greatly increasing the computational efficiency of such simulations while allowing the estimation of radical production rates. Data for generating such a closure model could be obtained by solving the bubble dynamics equations for a large number of bubble trajectories in hydrodynamic cavitation reactors operated under different conditions. The equilibrium reaction modelling approach, as recommended by this study, should be used to manage the computational cost of simulating the large number of trajectories. The development of such closure models will be the subject of future studies.

## CRediT authorship contribution statement

**Suat Canberk Ozan:** Conceptualization, Methodology, Software, Validation, Formal analysis, Investigation, Resources, Writing - original draft, Writing - review & editing, Visualization, Supervision, Project administration. **Pascal Jan Muller:** Methodology, Software, Validation, Formal analysis, Investigation, Writing - original draft. **Jan Hendrik Cloete:** Conceptualization, Methodology, Formal analysis, Resources, Writing - review & editing, Supervision, Project administration, Funding acquisition.

## Declaration of Competing Interest

The authors declare that they have no known competing financial interests or personal relationships that could have appeared to influence the work reported in this paper.

## Appendix A

### A.1 Temperature dependencies of physical properties

The reactive single bubble dynamics model includes mass and energy balances, Eqs. (3), (8), (9), that requires estimation of physical properties, such as heat capacity and molecular diffusivity, as a function of temperature. This subsection is dedicated to the details behind these calculations. For the phase change equations, given for water molecules in Eq. (3) and for reaction products in Eq. (8), the molecular diffusivity of the respective molecule is needed. To that extent, the Fuller et al. Fuller et al. (1966) correlation is employed.

The energy balance, Eq. (9), requires closures for the specific internal energy, *e*, and the enthalpy, *h*, of water and reaction products, the heat capacities, $c_{p}$ and $c_{v}$, of the gas mixture in the bubble, as well as the mixture’s thermal conductivity, λ. The specific internal energy of water, $e_{w}$, is determined through [23]

(18) $$e_{w}=R_{g}T_{B}\left(3+\sum \frac{\theta_{i}/T_{B}}{\exp\left(\theta_{i}/T_{B}\right)-1}\right)$$

where the characteristic vibrational temperatures for water are $\theta_{i}=2295K,\;5255K,\;5400K$ and $R_{g}$ is the universal gas constant. As the liquid phase is assumed to be a large body with a constant temperature $T_{L}$, the enthalpy of the evaporating water molecules can be taken as a constant given by [23], [30]

(19) $$h_{w}=4R_{g}T_{L}$$

The specific energy and the enthalpy of the reaction products are determined via the open-source software Cantera [36].

The heat capacities, $c_{p}$ and $c_{v}$, for water and argon are given by [23]

(20) $$c_{p,\mathrm{ar}}=c_{v,\mathrm{ar}}-N_{\mathrm{ar}}R_{g}=\frac{3}{2}N_{\mathrm{ar}}R_{g}$$

and

(21) $$c_{p,w}=c_{v,w}-N_{w}R_{g}=\left(3+\sum \frac{{\left(\theta_{i}/2T\right)}^{2}}{\sinh^{2}\left(\theta_{i}/2T\right)}\right)$$

Notice that the heat capacities are not specific and correspond to total heat capacity of a given compound in the bubble. The heat capacities for other species are determined through Cantera. The mixture $c_{p}$ and $c_{v}$ are then the summation of each component’s contribution. The mixture thermal conductivity is determined in a similar fashion by estimating the individual contribution from each species through Cantera. Finally, the thermal diffusivity is given as

(22) $$\kappa =\frac{\lambda V_{B}}{c_{p}}$$

as $c_{p}$ is the cumulative heat capacity and not the specific one. Here $V_{B}$ denotes the bubble volume.
